# Genetic predispositions to psychiatric disorders and the risk of COVID-19

**DOI:** 10.1186/s12916-022-02520-z

**Published:** 2022-08-23

**Authors:** Wenwen Chen, Yu Zeng, Chen Suo, Huazhen Yang, Yilong Chen, Can Hou, Yao Hu, Zhiye Ying, Yajing Sun, Yuanyuan Qu, Donghao Lu, Fang Fang, Unnur A. Valdimarsdóttir, Huan Song

**Affiliations:** 1 West China Biomedical Big Data Center, West China Hospital, Sichuan University, Chengdu, China; 2 Med-X Center for Informatics, Sichuan University, Chengdu, China; 3 Division of Nephrology, Kidney Research Institute, State Key Laboratory of Biotherapy and Cancer Center, West China Hospital, Sichuan University, Chengdu, China; 4 Department of Epidemiology & Ministry of Education Key Laboratory of Public Health Safety, School of Public Health, Fudan University, Shanghai, China; 5 Institute of Environmental Medicine, Karolinska Institute, Stockholm, Sweden; 6 Department of Epidemiology, Harvard T H Chan School of Public Health, Boston, MA USA; 7 Center of Public Health Sciences, Faculty of Medicine, University of Iceland, Reykjavík, Iceland; 8 Department of Medical Epidemiology and Biostatistics, Karolinska Institutet, Stockholm, Sweden

**Keywords:** Genetic predisposition, Psychiatric disorders, COVID-19

## Abstract

**Background:**

Whether a genetic predisposition to psychiatric disorders is associated with coronavirus disease 2019 (COVID-19) is unknown.

**Methods:**

Our analytic sample consisted of 287,123 white British participants in UK Biobank who were alive on 31 January 2020. We performed a genome-wide association study (GWAS) analysis for each psychiatric disorder (substance misuse, depression, anxiety, psychotic disorder, and stress-related disorders) in a randomly selected half of the study population (“base dataset”). For the other half (“target dataset”), the polygenic risk score (PRS) was calculated as a proxy of individuals’ genetic predisposition to a given psychiatric phenotype using discovered genetic variants from the base dataset. Ascertainment of COVID-19 was based on the Public Health England dataset, inpatient hospital data, or death registers in UK Biobank. COVID-19 cases from hospitalization records or death records were considered “severe cases.” The association between the PRS for psychiatric disorders and COVID-19 risk was examined using logistic regression. We also repeated PRS analyses based on publicly available GWAS summary statistics.

**Results:**

A total of 143,562 participants (including 10,868 COVID-19 cases) were used for PRS analyses. A higher genetic predisposition to psychiatric disorders was associated with an increased risk of any COVID-19 and severe COVID-19. The adjusted odds ratio (OR) for any COVID-19 was 1.07 (95% confidence interval [CI] 1.02–1.13) and 1.06 (95% CI 1.01–1.11) among individuals with a high genetic risk (above the upper tertile of the PRS) for substance misuse and depression, respectively, compared with individuals with a low genetic risk (below the lower tertile). Slightly higher ORs were noted for severe COVID-19, and similar result patterns were obtained in analyses based on publicly available GWAS summary statistics.

**Conclusions:**

Our findings suggest a potential role of genetic factors in the observed phenotypic association between psychiatric disorders and COVID-19. Our data underscore the need for increased medical surveillance for this vulnerable population during the COVID-19 pandemic.

**Supplementary Information:**

The online version contains supplementary material available at 10.1186/s12916-022-02520-z.

## Background

With over 518 million infected people and over 6 million related deaths, the coronavirus disease 2019 (COVID-19) pandemic, caused by the severe acute respiratory syndrome coronavirus 2 (SARS-CoV-2) infection, has led to an unprecedented crisis worldwide [1]. Evidence suggests that individuals have varying propensities for being infected with SARS-CoV-2 and that infected patients have heterogeneous outcomes [2]. Therefore, the identification of populations with increased susceptibility to COVID-19, especially those prone to a severe disease course, is critical for optimizing preventive measures.

Previous studies have reported an increased risk of infections (including life-threatening infections) among individuals with psychiatric disorders [3, 4]. Likewise, after the COVID-19 outbreak, accumulating evidence revealed that psychiatric disorders [5, 6], such as depression and schizophrenia, were also associated with an increased risk of COVID-19, possibly through mechanisms similar to those leading to other infections [6]. Besides the immune dysfunction observed widely among individuals with a psychiatric illness [7], other explanations might include an unhealthy lifestyle, such as tobacco smoking and physical inactivity [8]. Furthermore, one recent investigation, which suggested a shared genetic vulnerability to psychiatric disorders and infections, reported a strong genetic association between having at least one psychiatric diagnosis and infection [9]. However, to our knowledge, no study, so far, has explored whether genetic predispositions to psychiatric disorders contribute to susceptibility to COVID-19, especially the severe form of the disease course. Furthermore, due to similarities in protein architecture and pathogenicity between SARS-CoV-2 and other coronaviruses [10], the findings of the present study may be relevant for other infections in terms of recognizing and optimizing preventative measures in high-risk populations.

Based on the results of genome-wide association studies (GWAS), a polygenic risk score (PRS), or the sum of all risk alleles weighted by the effect size of each variant, can be generated. The PRS represents an individual’s overall genetic risk for a given disease, such as a psychiatric disorder. It can further be used to predict the risk of developing a second disease outcome and thereby illustrating the genetic association between a disease pair [11, 12].

As a continuation of our previous research, which demonstrated a robust association between pre-pandemic psychiatric disorders and COVID-19 risk [6], here, we aimed to explore potential underlying mechanisms by testing if a genetic predisposition to psychiatric disorders is associated with a risk of SARS-CoV-2 infection and progressive COVID-19 using the UK Biobank databases.

## Methods

### Study design

#### UK Biobank

Our study was based on data from the large-scale prospective cohort of UK Biobank, which enrolled 502,507 individuals aged between 40 and 69 years across the UK during 2006–2010 (i.e., ages 50–83 years at the time of the COVID-19 outbreak). Genotyping data were obtained from 488,377 blood samples collected at baseline for each participant. They were assayed using UK BiLEVE (Applied Biosystems, Foster City, CA, USA) and UK Biobank Axiom Array [13]. After quality control following the UK Biobank pipeline, genotype imputation was completed using the Haplotype Reference Consortium and the UK10K haplotype resource as reference panels [13]. The kinship coefficient and principal components (PCs), which were calculated using the KING tool, were also provided by UK Biobank. Details about the quality-control pipeline of UK Biobank and imputation methods have been described previously [13]. The final quality-controlled and imputed-genotype dataset was the basis for our analyses and contained >93 million autosomal single-nucleotide polymorphisms (SNPs) of 487,296 individuals.

Phenotypic data such as sex and birth year were collected upon recruitment using a questionnaire. Health-related outcomes were obtained through periodically linked data from multiple national datasets, including death registries and inpatient hospital data from across England, Scotland, and Wales [13]. After the global outbreak of COVID-19, UK Biobank was also been linked to Public Health England (PHE), where results of COVID-19 tests by reverse transcription-polymerase chain reaction (RdRp gene assay) from oral swabs have been documented since 16 March 2020 [14].

UK Biobank collected all data after written informed consent had been obtained from each participant. The study protocol had full ethical approval (16/NW/0274) from the National Research Ethics Service of the UK National Health Service. The present study was also approved (2020.661) by the biomedical research ethics committee of West China Hospital (Chengdu, China).

#### Ascertainment of psychiatric disorders and COVID-19

To align with our previous analyses of phenotypic association [6], we used an identical approach for ascertaining psychiatric disorders and COVID-19 in the present study. Briefly, we defined five broad diagnostic categories of psychiatric disorders (substance misuse, depressive disorders, anxiety disorders, psychotic disorders, and stress-related disorders) based on hospital admissions. The diagnosis of these disorders was according to the International Classification of Diseases, Tenth Revision (ICD-10), or ICD-9 codes in the UK Biobank inpatient hospital data (Additional file 1: Table S1) before 31 January 2020. Identification of COVID-19 was based on a positive test result from the PHE dataset, a diagnosis in the UK Biobank inpatient hospital data, or a cause of death based on the UK Biobank mortality data (ICD codes are shown in Additional file 1: Table S1), updated until 18 October 2021. COVID-19 cases from hospitalization records or death records were considered “severe cases.”

#### PRS for psychiatric disorders

We conducted genetic analyses of white British participants in UK Biobank who were registered in England at recruitment, alive, and trackable on 31 January 2020 and had available genetic data (*n* = 346,502). Standard quality control of GWAS was carried out. Briefly, we restricted our analyses to autosomal biallelic SNPs and removed variants with a call rate <98%, minor allele frequency <0.01, or deviation from Hardy–Weinberg equilibrium (*p* < 10^−6^). Then, related individuals, up to the third degree (i.e., kinship coefficient > 0.044) [15], were removed before GWAS based on the principle of prioritizing the inclusion of individuals with COVID-19. We also removed individuals having a genotyping rate < 98% and outlier samples based on their abnormal heterozygosity level, leaving 287,123 participants for further analysis. The details of the quality-control strategy are summarized in Additional file 1: Fig. S1.

**Fig. 1:**
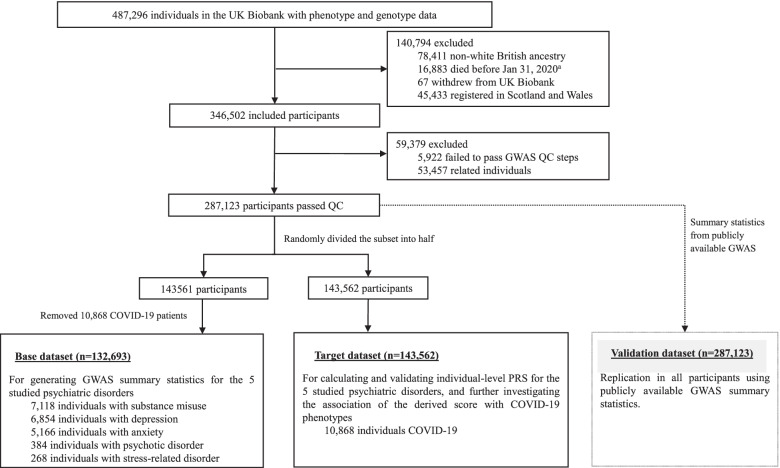
Study design. ^a^The first COVID-19 case was diagnosed on Jan 31, 2020, in the UK

We calculated the PRS for each studied psychiatric disorder based on two types of GWAS summary statistics. First, due to human heterogeneity (and therefore possible limited portability of the PRS between populations [16]) and to keep consistent ascertainment of psychiatric disorders between the present study and our previous study on phenotypic association [6], we undertook a GWAS by splitting the data from UK Biobank into a base dataset and target dataset (see the study design in Fig. 1). GWAS analyses of the base dataset (i.e., 50% of participants selected randomly from the study population) were performed after removal of all individuals with confirmed COVID-19 (*n* = 10,868) to avoid the influence of phenotypic associations (e.g., between depression and COVID-19) on the identification of the genetic background for the exposure trait (e.g., depression). Specifically, the associations between these phenotypes (as binary variables) and each eligible SNP were tested using logistic regression models adjusted for the covariates sex, birth year, genotyping array, and the top-10 PCs. Second, given that publicly available GWAS of the five specific psychiatric disorders have larger discovery samples, in the dataset that included all eligible and unrelated participants (*n* = 287,123), we also generated PRSs for these psychiatric disorders based on summary statistics from publicly available GWAS [17–21].

The PRS for each exposure trait was calculated as the weighted sum of the risk alleles based on the summary statistics (i.e., effect sizes and standard errors for the variants) of the GWAS results mentioned above using the least absolute shrinkage and selection operator (LASSO) approach, which allowed heavy shrinkage in the effect estimates of SNPs via regularization methods [22]. The hg19 genome of the European population was used as a reference panel to handle linkage disequilibrium (LD) [23].

### Statistical analyses

First, we validated the predictability of the generated PRSs (i.e., PRS calculated based on GWAS from UK Biobank and that based on publicly available GWAS) on the corresponding category of psychiatric disorder in UK Biobank data using logistic regression models adjusted for sex, birth year (1936–1940, 1941–1945, 1946–1950, 1951–1955, 1956–1960, 1961–1965, and 1966–1970), genotyping array, and significant PCs for population heterogeneity (i.e., *p* < 0.05). The variance explained by the PRS was assessed as the difference in variance from the full model, including the PRS compared with the basic model, and represented as Nagelkerke’s *R*^2^.

Then, we examined the association between the PRS of a specific category of psychiatric disorder and the risk of COVID-19, as well as severe COVID-19, using odds ratios (ORs) with 95% confidence intervals (CIs) derived from logistic regression models adjusted for the covariates mentioned above. In addition to treating the standardized PRS as a continuous variable, we also divided participants into “low,” “moderate,” and “high” genetic risk groups based on the tertile distribution of the PRS and then compared the risk of COVID-19 outcomes using the group of low genetic risk (i.e., below the lower tertile of the PRS) as the reference. The degree of pleiotropy or genetic correlation between psychiatric disorders and COVID-19 was additionally estimated using cross-trait LD score regression based on publicly available GWAS summary statistics of both traits [17–21, 24].

In sensitivity analyses, all the analyses stated above were repeated after the application of the standard clumping + thresholding (C+T) approach, instead of the LASSO approach used in the main analyses, for PRS calculation. This method handles the shrinkage-of-effect size estimates through selectively including SNPs with a GWAS association *p*-value below a certain threshold (e.g., *p* < 5 × 10^−8^). Then, through clumping, only SNPs that were largely independent of each other were retained so that their effects could be summed. We computed the PRS under 10 *p-*value thresholds (i.e., 5 × 10^−8^, 1 × 10^−6^, 1 × 10^−4^, 1 × 10^−3^, 0.05, 0.1, 0.2, 0.3, 0.4, and 0.5).

PLINK (version 1.9) and R software (version 4.0) were used for GWAS and PRS calculations (“LASSO” package, version 0.4.4) [22]. LD score regression was done by LDSC software (version 1.0.1) [25]. Other analyses were carried out with R software (version 4.0), and *p* < 0.05 (two-sided) was considered significant.

## Results

In total, 287,123 participants (Fig. 1) were included for analyses with a mean age of 68.07 years at the time of the COVID-19 outbreak. Of these, 130,896 (45.59%) were men. Among the 132,693 participants in the base dataset, there were 7118 (5.36%), 6854 (5.17%), 5166 (3.89%), 384 (0.29%), and 268 (0.20%) cases of substance misuse, depression, anxiety, psychotic disorder, and stress-related disorders, respectively. The target dataset had 143,562 participants, including 10,868 COVID-19 cases, among which 1582 were hospitalized for COVID-19 or died due to COVID-19 (severe cases).

Based on the base dataset of UK Biobank, GWAS results were summarized using a Manhattan plot (Additional file 1: Fig. S2). In brief, the PRSs (as continuous variables) were significantly associated with an increased risk of the corresponding psychiatric disorders in the target dataset (Additional file 1: Table S2). However, the highest variance explained by an increase of one standard deviation (SD) of PRSs was moderate, with ORs ranging between 1.11 (95% CI 1.08–1.14) and 1.20 (95% CI 1.09–1.33). The predictability of the PRS calculated based on publicly available GWAS was, in general, comparable with that generated based on GWAS from UK Biobank (Additional file 1: Table S2).

**Fig. 2:**
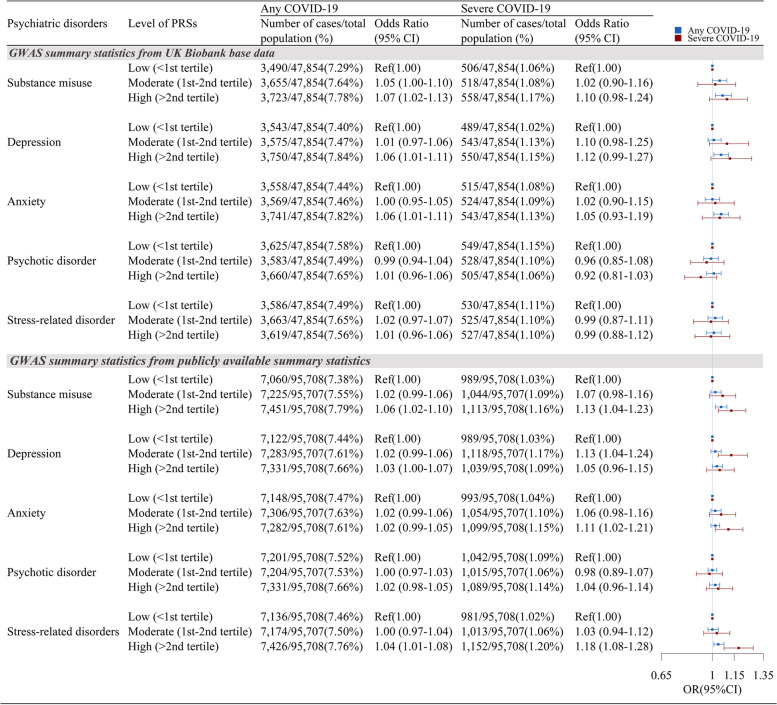
The association between different levels of polygenic risk scores (PRSs) for psychiatric disorders and COVID-19 risk. Odds ratio and 95% confidence interval were estimated by logistic regression models, adjusting for birth year, sex, genotyping array, and significant ancestry principal components (for any COVID-19: PC5, PC9, PC23, PC25, PC26, and PC27; for severe COVID-19: PC1, PC5, PC9, PC25, PC31, and PC39)

Using the PRS as a proxy for a genetic predisposition to a given psychiatric disorder, we examined the association between the genetic risk of a psychiatric disorder and the risk of COVID-19 and severe COVID-19. Using PRSs based on UK Biobank GWAS, we obtained increased risks of any COVID-19 in relation to an increase of 1SD in the PRSs of substance misuse, depression, and anxiety (Table 1), indicating a 3% (95% CI 1–5%) increased risk of any COVID-19 per 1-SD increase of the corresponding PRS after adjustment for all covariates. Analyses of categorized PRSs revealed similar results (Fig. 2), which showed an increase in COVID-19 risk with increased genetic susceptibility to substance misuse, depression, and anxiety. For instance, compared with individuals with a low genetic risk of substance misuse (PRS < lower tertile), the adjusted OR was 1.05 (95% CI 1.00–1.10) and 1.07 (95% CI 1.02–1.13) for individuals with moderate (PRS = lower–upper tertile) and high genetic risk (PRS >upper tertile) of substance misuse, respectively. For severe COVID-19 (which had lower precision due to fewer cases), these observed associations seemed to be enhanced further. For instance, a 1-SD increase in the PRS of depression was marginally associated with a 5% (OR = 1.05; 95% CI 0.99–1.10) increased risk of severe COVID-19 (Table 1). The OR was 1.12 (95% CI 0.99–1.27) among individuals with a high genetic risk of depression compared with those with a low genetic risk of depression (Fig. 2).

**Table 1:** The association between polygenic risk scores (PRSs) for psychiatric disorders and COVID-19

Psychiatric disorders	Any COVID-19		Severe COVID-19	
	Number of cases/total population (%)	Odds ratio	Number of cases/total population (%)	Odds ratio
		(95% confidence interval)^**a**^		(95% confidence interval)^**a**^
***GWAS summary statistics from UK Biobank base data***				
**Substance misuse**	10,868/143,562 (7.57%)	1.03 (1.01–1.05)	1,582/143,562 (1.10%)	1.05 (1.00–1.10)
**Depression**		1.03 (1.01–1.05)		1.05 (0.99–1.10)
**Anxiety**		1.03 (1.01–1.05)		1.02 (0.97–1.07)
**Psychotic disorder**		1.00 (0.98–1.02)		0.96 (0.91–1.01)
**Stress-related disorder**		1.00 (0.98–1.02)		1.01 (0.96–1.06)
***GWAS summary statistics from publicly available GWAS summary statistics***				
**Substance misuse**	21,736/287,123 (7.57%)	1.03 (1.01–1.04)	3,146/287,123 (1.10%)	1.05 (1.01–1.08)
**Depression**		1.02 (1.00–1.03)		1.03 (1.00–1.07)
**Anxiety**		1.01 (1.00–1.03)		1.05 (1.01–1.08)
**Psychotic disorder**		1.01 (0.99–1.02)		1.01 (0.98–1.05)
**Stress-related disorder**		1.02 (1.01–1.03)		1.08 (1.04–1.11)

^a^Odds ratio and 95% confidence interval (an increase of one standard deviation in the corresponding PRS) were estimated by logistic regression models, adjusting for birth year, sex, genotyping array, and significant ancestry principal components (for any COVID-19: PC5, PC9, PC23, PC25, PC26, and PC27, and for severe COVID-19: PC1, PC5, PC9, PC25, PC31, and PC39)

Furthermore, the PRSs generated from publicly available GWAS summary statistics yielded largely similar result patterns (Table 1 and Fig. 2). However, an excess risk of any COVID-19, as well as severe COVID-19, in relation to an increased PRS for stress-related disorders (OR = 1.02, 95% CI 1.01–1.03 and OR = 1.08, 95% CI 1.04–1.11 for any COVID-19 and severe COVID-19, respectively, per 1-SD increase of PRS) was observed, which was considered a null association with COVID-19 based on our UK Biobank GWAS.

The results of LD score regression corroborated the findings of the PRS analyses, indicating a genetic overlap (i.e., shared genetic components) between substance misuse and COVID-19 (*r* for genetic = 0.22, 95% CI 0.04–0.41, *p* = 0.0148), as well as between depression and COVID-19 (*r* for genetic = 0.13, 95% CI 0.04–0.22, *p* = 0.005) (Table 2).

**Table 2:** Genetic correlation between psychiatric disorders and COVID-19 assessed by LD score regression

Psychiatric disorders^**a**^	Any COVID-19^**b**^		Inpatient COVID-19^**b**^	
	rg (95% confidence interval)^**c**^	***p***	rg (95% confidence interval)^**c**^	***p***
**Substance misuse**	0.22 (0.04–0.41)	0.01	0.13 (−0.03–0.30)	0.10
**Depression**	0.13 (0.04–0.22)	5.00 × 10^−3^	0.18 (0.09–0.27)	9.82 × 10^−5^
**Anxiety**	0.07 (−0.18–0.32)	0.57	0.08 (−0.14–0.30)	0.49
**Psychotic disorder**	−0.03 (−0.10–0.04)	0.42	0.02 (−0.04–0.08)	0.58
**Stress-related disorder**	0.10 (−0.07–0.26)	0.25	0.11 (−0.02–0.24)	0.09

*rg r* for genetic

^a^GWAS summary statistics for psychiatric disorders were obtained from publicly available GWAS

^b^GWAS summary statistics for COVID-19 were obtained from https://www.covid19hg.org/results/r7/

^c^Genetic correlations were assessed by LD score regression

In sensitivity analyses, the PRSs calculated using the C+T method revealed comparable prediction accuracy for the studied psychiatric traits as those generated by LASSO (Additional file 1: Tables S3–S7). Using the *p*-value threshold with the highest *R*^*2*^ for its regression model (i.e., interpreting as the one with the largest variance explained), we observed largely identical estimates as those in the main analyses (Additional file 1: Table S8 and Additional file 1: Fig. S3). We have also listed the estimates under different *p*-value thresholds in Additional file 1: Table S9.

## Discussion

Based on a large-scale prospective cohort of UK Biobank with comprehensive phenotypic and genetic information, we demonstrated that genetic predispositions to various psychiatric disorders (including depression, substance misuse, and anxiety) were associated with a risk of COVID-19, including severe COVID-19, among a population aged >50 years who are highly vulnerable to such an infectious disease. To our knowledge, this is the first study, to date, that has examined the relationship of the genetic basis of psychiatric disorders with COVID-19 using individual-level genotyping data. Our findings aid the interpretation of the reported phenotypic association of pre-pandemic psychiatric disorders with COVID-19 risk by demonstrating a role of an underlying genetic mechanism in this link. The genetically driven susceptibility to COVID-19 (especially to severe and fatal COVID-19) among individuals prone to psychiatric disorders underscores the need for heightened clinical awareness and medical care for this vulnerable population during the COVID-19 pandemic. Further studies are, however, warranted to verify our findings and to explore functional mechanisms.

The hypothesis that a pre-existing psychiatric disorder may influence susceptibility to infectious diseases has long been discussed. Many previous studies have consistently reported an increased risk of infection, including pneumonia [26], sepsis [27], and other life-threatening infections [3], subsequent to a psychiatric illness, particularly stress reactions and related psychiatric disorders. The proposed mechanisms for the observed associations include dysregulation of the immune response, an inflammatory profile [28], and physiological alterations that commonly accompany a psychiatric presentation. Changes in lifestyle, such as tobacco smoking and alcohol abuse [8], following psychiatric disorders may also contribute to an altered susceptibility to infections. Recently, a national study in Denmark suggested a genetic component in the association between psychiatric disorders and infections by demonstrating a strong genetic link between having at least one psychiatric diagnosis and infection [9]. Furthermore, a GWAS analysis found that schizophrenia-associated genetic factors have important roles in immunity, providing support for the speculated genetic link between the immune system and schizophrenia [18].

Accumulating evidence, including our previous work [6], has revealed that psychiatric disorders are associated with an elevated risk of COVID-19 and its related hospitalizations and deaths [5]. Furthermore, an excess risk of hospitalization for other infections has been observed at a comparable level to that of COVID-19 among individuals with pre-existing psychiatric disorders during the COVID-19 outbreak [6]. Therefore, it is rational to speculate that the increased susceptibility to COVID-19 and other infections may have shared etiologies, such as dysfunction of the immune system [6]. Indeed, a meta-analysis of GWAS for COVID-19 identified several loci that have been associated previously with autoimmune and inflammatory diseases [29]. Also, increased levels of cytokines have been reported to be correlated with disease deterioration and fatal COVID-19 [30], which implies a role of altered immune responses in disease progression. Our study provides supporting evidence of a genetic association between several psychiatric disorders and COVID-19. This observation is in accordance with the findings of a study that revealed a positive genetic correlation between cannabis-use disorder and COVID-19 hospitalization using analyses of LD score regression of summarized GWAS results [31]. A considerable proportion of the general population has a history of psychiatric disorders (17–29%) [32]. The prevalence is even higher (46%) in the elderly [33], who are also more vulnerable to COVID-19. Therefore, our findings reveal the necessity of improved surveillance and further exploration of possible interventions to reduce the risk of COVID-19 and disease deterioration among this vulnerable group.

The merits of the present study include the application of the PRS to assess the association between the genetic risk of psychiatric disorders and COVID-19 in a large population. This strategy enabled the identification of a dose–response relationship between the PRS for different psychiatric traits and COVID-19 (especially severe COVID-19). These findings corroborate the previously observed phenotypic associations between psychiatric disorders and COVID-19 and shed light on the underlying mechanisms of such associations. In addition, because population heterogeneity may undermine the prediction accuracy of a PRS due to not only the different ancestries but also differences in the characteristics of populations (such as differences in sex, age, or socioeconomic status [16]), we initially used a base dataset to calculate the PRS of each psychiatric disorder and then employed PRSs to test genetic associations using a target dataset. Similar methods have been applied to investigate an association of genetic predisposition to asthma with COVID-19 [11]. Furthermore, given that similar analyses using PRSs generated based on publicly available GWAS corroborated the results of the PRS analyses based on the GWAS of the UK Biobank sample, our findings seem robust with respect to the choice of GWAS summary statistics.

Our study had four main limitations. First, the genetic association between stress-related disorders and COVID-19 remains inclusive as different results were obtained using PRSs based on GWAS from UK Biobank and those generated from GWAS of the population-based Lundbeck Foundation Initiative for Integrative Psychiatric Research (iPSYCH) study [21]. However, the GWAS of stress-related disorders has limited sample sizes. Our base dataset from UK Biobank comprised 268 cases with a stress-related disorder, whereas the iPSYCH study involved 9831 cases. Therefore, the genetic profile of stress-related disorders is not well established, and whether the genetic predisposition to a stress-related disorder is associated with COVID-19 risk must be studied further. Second, in selecting SNPs for PRS construction using the C+T approach, relatively loose *p*-value thresholds were applied, possibly resulting in “noise” from redundant loci, and its impact on the observed associations is unclear. Third, UK Biobank is not a representative sample of the entire UK population, as it recruited only 5.5% of the target population, and the participants were predominately white (94%), were more likely living in less socioeconomically deprived areas than non-participants, and had fewer self-reported health conditions than the general population [34]. Also, the participants were relatively older (50–83 years) at the time of the COVID-19 outbreak. Thus, generalization of our findings to the younger or entire UK population, as well as other populations, must be made with caution. Fourth, given that the PRS contains information only from relatively common variants, the impact of rare and low-frequency genetic variants merits further investigation.

## Conclusions

In the UK Biobank population, we found genetic predispositions to several psychiatric disorders were associated with an increased risk of COVID-19, which may partially explain the observed phenotypic associations between psychiatric disorders and COVID-19. Notably, the gene-driven susceptibility to COVID-19 among individuals prone to psychiatric disorders underscores the need for extra awareness and medical care for this vulnerable population. Further studies are needed to identify particular genetic variants with the aim of understanding the underlying mechanisms, optimizing risk stratification, and providing molecular targets for COVID-19 prevention.

## Supplementary Information

**Additional file 1: Table S1.** International Classification of Disease (ICD) codes, ninth (ICD-9) and tenth (ICD-10) revisions for diagnoses used in this study. **Table S2.** The association between polygenic risk scores (PRSs, calculated using LASSO approach) for varied psychiatric disorders and its corresponding psychiatric disorders. **Table S3.** Associations between polygenic risk scores (PRS, calculated using Clumping + Thresholding approach) for substance misuse and the risk of substance misuse at different p value thresholds. **Table S4.** Associations between polygenic risk scores (PRS, calculated using Clumping + Thresholding approach) for depression and the risk of depression at different p value thresholds. **Table S5.** Associations between polygenic risk scores (PRS, calculated using Clumping + Thresholding approach) for anxiety and the risk of anxiety at different p value thresholds. **Table S6.** Associations between polygenic risk scores (PRS, calculated using Clumping + Thresholding approach) for psychotic disorder and the risk of psychotic disorder at different p value thresholds. **Table S7.** Associations between polygenic risk scores (PRS, calculated using Clumping + Thresholding approach) for stress-related disorder and the risk of stress-related disorder at different p value thresholds. **Table S8.** The association between polygenic risk scores (PRSs, calculated using Clumping + Thresholding approach) for psychiatric disorders and COVID-19. **Table S9.** The association between polygenic risk scores (PRSs, calculated using Clumping + Thresholding approach) for psychiatric disorders under different p value thresholds and COVID-19. **Figure S1.** Flowchart of the entire GWAS quality control process. **Figure S2.** Manhattan of the SNP-Based GWAS of psychiatric disorders, using UK Biobank base data (SNP=single-nucleotide polymorphism). **Figure S3.** The association between categorized polygenic risk scores (PRSs, calculated using Clumping + Thresholding approach) for psychiatric disorders and COVID-19 risk^a^.

## Data Availability

Data from the UK Biobank (http://www.ukbiobank.ac.uk/) are available to all researchers upon making an application. Part of this research was conducted using the UK Biobank Resource under Application 54803 (approved on October 29, 2019).

## Abbreviations

CI: Confidence interval
COVID-19: Coronavirus disease 2019
GWAS: Genome-wide association studies
HRC: Haplotype Reference Consortium
ICD-10: International Classification of Diseases 10th edition
OR: Odds ratio
PHE: Public Health England
PRSs: Polygenetic risk scores
SARS-CoV-2: Severe acute respiratory syndrome coronavirus 2
SNPs: Autosomal single-nucleotide polymorphisms
TDI: Townsend deprivation index
