# Supplementary material for: Genetic predispositions to psychiatric disorders and the risk of COVID-19
Source: BMC Med. 2022 Aug 23;20:314. doi: 10.1186/s12916-022-02520-z (PMC9397166; doi:10.1186/s12916-022-02520-z)
Supplement: Supplementary file 1 — Additional file 1: Table S1. International Classification of Disease (ICD) codes, ninth (ICD-9) and tenth (ICD-10) revisions for diagnoses used in this study. Table S2. The association between polygenic risk scores (PRSs, calculated using LASSO approach) for varied psychiatric disorders and its corresponding psychiatric disorders. Table S3. Associations between polygenic risk scores (PRS, calculated using Clumping + Thresholding approach) for substance misuse and the risk of substance misuse at different p value thresholds. Table S4. Associations between polygenic risk scores (PRS, calculated using Clumping + Thresholding approach) for depression and the risk of depression at different p value thresholds. Table S5. Associations between polygenic risk scores (PRS, calculated using Clumping + Thresholding approach) for anxiety and the risk of anxiety at different p value thresholds. Table S6. Associations between polygenic risk scores (PRS, calculated using Clumping + Thresholding approach) for psychotic disorder and the risk of psychotic disorder at different p value thresholds. Table S7. Associations between polygenic risk scores (PRS, calculated using Clumping + Thresholding approach) for stress-related disorder and the risk of stress-related disorder at different p value thresholds. Table S8. The association between polygenic risk scores (PRSs, calculated using Clumping + Thresholding approach) for psychiatric disorders and COVID-19. Table S9. The association between polygenic risk scores (PRSs, calculated using Clumping + Thresholding approach) for psychiatric disorders under different p value thresholds and COVID-19. Figure S1. Flowchart of the entire GWAS quality control process. Figure S2. Manhattan of the SNP-Based GWAS of psychiatric disorders, using UK Biobank base data (SNP=single-nucleotide polymorphism). Figure S3. The association between categorized polygenic risk scores (PRSs, calculated using Clumping + Thresholding approach) for psychiatric disorders [file 12916_2022_2520_MOESM1_ESM.doc]

**Genetic predispositions to** **psychiatric disorders and the risk of COVID-19**

**Additional file 1**

Wenwen Chen; Yu Zeng; Chen Suo; Huazhen Yang; Yilong Chen; Can Hou; Yao Hu; Zhiye Ying; Yajing Sun; Yuanyuan Qu; Donghao Lu; Fang Fang; Unnur A. Valdimarsdóttir; Huan Song

**Content**

**Table S1** International Classification of Disease (ICD) codes, ninth (ICD-9) and tenth (ICD-10) revisions for diagnoses used in this study

**Table S2** The association between polygenic risk scores (PRSs, calculated using LASSO approach) for varied psychiatric disorders and its corresponding psychiatric disorders

**Table S3** Associations between polygenic risk scores (PRS, calculated using Clumping + Thresholding approach) for substance misuse and the risk of substance misuse at different p value thresholds

**Table S4** Associations between polygenic risk scores (PRS, calculated using Clumping + Thresholding approach) for depression and the risk of depression at different p value thresholds

**Table S5** Associations between polygenic risk scores (PRS, calculated using Clumping + Thresholding approach) for anxiety and the risk of anxiety at different p value thresholds

**Table S6** Associations between polygenic risk scores (PRS, calculated using Clumping + Thresholding approach) for psychotic disorder and the risk of psychotic disorder at different p value thresholds

**Table S7** Associations between polygenic risk scores (PRS, calculated using Clumping + Thresholding approach) for stress-related disorder and the risk of stress-related disorder at different p value thresholds

**Table S8** The association between polygenic risk scores (PRSs, calculated using Clumping + Thresholding approach) for psychiatric disorders and COVID-19

**Table S9** The association between polygenic risk scores (PRSs, calculated using Clumping + Thresholding approach) for psychiatric disorders under different p value thresholds and COVID-19

**Figure S1** Flowchart of the entire GWAS quality control process

**Figure S2** Manhattan of the SNP-Based GWAS of psychiatric disorders, using UK Biobank base data (SNP=single-nucleotide polymorphism)

**Figure S3** The association between categorized polygenic risk scores (PRSs, calculated using Clumping + Thresholding approach) for psychiatric disorders and COVID-19 risk ^a^

**Table S1 International Classification of Disease (ICD) codes, ninth (ICD-9) and tenth (ICD-10) revisions for diagnoses used in this study**

| **Diagnose** | **ICD-10** | **ICD-9** |
| --- | --- | --- |
| Substance misuse | F10-F19 | 291, 303-305 |
| Depression | F32-F33 | 2961, 3004, 311 |
| Anxiety | F40-F41 | 3000, 3002 |
| Psychotic disorder | F20-F29 | 295, 297, 298 |
| Stress-related disorder | F43 | 308, 309 |
| COVID-19 | U07.1, U07.2 | - |

**Table S2 The association between polygenic risk scores (PRSs, calculated using LASSO approach) for varied psychiatric disorders and its corresponding psychiatric disorders**

| Psychiatric disorders | **GWAS summary statistics from UK Biobank base data** | | **Publicly available GWAS summary statistics** | |
| --- | --- | --- | --- | --- |
|  | **Odds Ratio**  **(95% confidence interval) ^a^** | R^2^ | **Odds Ratio**  **(95% confidence interval) ^a^** | R^2^ |
| Substance misuse | 1.17 (1.14-1.20) | 2.50% | 1.06 (1.04-1.08) | 2.06% |
| Depression | 1.13 (1.11-1.16) | 0.96% | 1.24 (1.22-1.26) | 1.36% |
| Anxiety | 1.11 (1.08-1.14) | 1.15% | 1.08 (1.06-1.10) | 1.14% |
| Psychotic disorder | 1.20 (1.09-1.33) | 1.33% | 1.81 (1.68-1.93) | 3.22% |
| Stress-related disorders | 1.14 (1.02-1.27) | 2.70% | 1.25 (1.16-1.36) | 2.04% |

a Odd Ratio and 95% CIs (per standard deviation increase in the corresponding PRS) were estimated by logistic regression models, adjusting for birth year, sex, genotyping array, and ancestry principal components. R^2^ was Nagelkerke’s squared (R square). *GWAS = Genome wide association study.

**Table S3 Associations between polygenic risk scores (PRS, calculated using Clumping + Thresholding approach) for substance misuse and the risk of substance misuse at different p value thresholds**

| Pt | **GWAS summary statistics from UK Biobank base data** | | | |  | **GWAS summary statistics from ICC** | | | |
| --- | --- | --- | --- | --- | --- | --- | --- | --- | --- |
|  | N_SNP | OR (95% CI) | R^2^ | p value |  | N_SNP | OR (95% CI) | R^2^ | p value |
| Pt5e-08 | - | - | - | - |  | - | - | - | - |
| Pt1e-06 | 1 | 1.02 (0.99-1.04) | 2.200% | 0.176 |  | 2 | 1.00 (0.99-1.02) | 2.021% | 0.57 |
| Pt1e-04 | 41 | 1.02 (1.00-1.05) | 2.204% | 0.037 |  | 79 | 1.02 (1.00-1.04) | 2.025% | 0.019 |
| Pt0.001 | 275 | 1.07 (1.04-1.09) | 2.249% | 1.63×10^-8^ |  | 639 | 1.01 (0.99-1.02) | 2.021% | 0.402 |
| Pt0.05 | 6724 | 1.13 (1.11-1.16) | 2.384% | 2.13×10^-26^ |  | 16859 | 1.04 (1.02-1.06) | 2.037% | 8.07×10^-6^ |
| Pt0.1 | 11155 | 1.14 (1.12-1.17) | 2.412% | 3.93×10^-30^ |  | 28634 | 1.04 (1.02-1.06) | 2.041% | 1.05×10^-6^ |
| Pt0.2 | 18103 | 1.14 (1.11-1.17) | 2.399% | 1.91×10^-28^ |  | 47172 | 1.04 (1.02-1.06) | 2.037% | 8.17×10^-6^ |
| Pt0.3 | 23492 | 1.14 (1.11-1.16) | 2.393% | 1.12×10^-27^ |  | 62092 | 1.04 (1.02-1.06) | 2.037% | 8.65×10^-6^ |
| Pt0.4 | 27922 | 1.14 (1.11-1.16) | 2.392% | 1.93×10^-27^ |  | 74199 | 1.04 (1.03-1.06) | 2.041% | 7.08×10^-7^ |
| Pt0.5 | 31519 | 1.13 (1.11-1.16) | 2.385% | 1.32×10^-26^ |  | 84034 | 1.04 (1.03-1.06) | 2.041% | 8.63×10^-7^ |

OR were adjusting for birth year, sex, genotyping array, and ancestry principal components. Pt was p value threshold. N_SNP was the number of SNP used predictive model. R^2^ was Nagelkerke’s squared (R square). PRS = polygenic risk score. GWAS = Genome wide association study. ICC = International Cannabis Consortium. – No results in this fields.

**Table S4 Associations between polygenic risk scores (PRS, calculated using Clumping + Thresholding approach) for depression and the risk of depression at different** **p value thresholds**

| Pt | **GWAS summary statistics from UK Biobank base data** | | | |  | **GWAS summary statistics from PGC** | | | |
| --- | --- | --- | --- | --- | --- | --- | --- | --- | --- |
|  | N_SNP | OR (95% CI) | R^2^ | p value |  | N_SNP | OR (95% CI) | R^2^ | p value |
| Pt5e-08 | - | - | - | - |  | 2 | 1.04 (1.02-1.06) | 0.844% | 5.45×10^-6^ |
| Pt1e-06 | 0 | 0.99 (0.97-1.01) | 0.781% | 0.33 |  | 13 | 1.04 (1.03-1.06) | 0.847% | 7.28×10^-7^ |
| Pt1e-04 | 43 | 1.01 (0.99-1.04) | 0.782% | 0.257 |  | 238 | 1.07 (1.06-1.09) | 0.886% | 3.27×10^-17^ |
| Pt0.001 | 283 | 1.05 (1.02-1.07) | 0.805% | 8.92×10^-5^ |  | 1235 | 1.12 (1.10-1.13) | 0.971% | 2.36×10^-39^ |
| Pt0.05 | 6741 | 1.10 (1.08-1.13) | 0.891% | 4.56×10^-16^ |  | 23444 | 1.17 (1.15-1.19) | 1.115% | 1.20×10^-76^ |
| Pt0.1 | 11220 | 1.10 (1.08-1.13) | 0.889% | 6.24×10^-16^ |  | 39265 | 1.17 (1.15-1.19) | 1.130% | 1.90×10^-80^ |
| Pt0.2 | 18186 | 1.10 (1.07-1.12) | 0.880% | 1.05×10^-14^ |  | 65251 | 1.17 (1.16-1.19) | 1.135% | 7.51×10^-82^ |
| Pt0.3 | 23679 | 1.10 (1.07-1.12) | 0.885% | 2.75×10^-15^ |  | 86591 | 1.18 (1.16-1.20) | 1.140% | 3.88×10^-83^ |
| Pt0.4 | 28035 | 1.10 (1.08-1.13) | 0.892% | 2.52×10^-16^ |  | 104582 | 1.18 (1.16-1.20) | 1.142% | 1.46×10^-83^ |
| Pt0.5 | 31630 | 1.10 (1.08-1.13) | 0.893% | 2.18×10^-16^ |  | 119974 | 1.18 (1.16-1.20) | 1.142% | 1.13×10^-83^ |

OR were adjusting for birth year, sex, genotyping array, and ancestry principal components. Pt was p value thresholds. N_SNP was the number of SNP in the predictive model. R^2^ was Nagelkerke’s squared (R square). PRS = polygenic risk score. GWAS = Genome wide association study. PGC = Psychiatric Genomics Consortium. – No results in this fields.

**Table S5 Associations between polygenic risk scores (PRS, calculated using Clumping + Thresholding approach) for anxiety and the risk of anxiety at different p value thresholds**

| Pt | **GWAS summary statistics from UK Biobank base data** | | | |  | **GWAS summary statistics from PGC** | | | |
| --- | --- | --- | --- | --- | --- | --- | --- | --- | --- |
|  | N_SNP | OR (95% CI) | R^2^ | p value |  | N_SNP | OR (95% CI) | R^2^ | p value |
| Pt5e-08 | - | - | - | - |  | 1 | 0.99 (0.97-1.01) | 1.082% | 0.297 |
| Pt1e-06 | 1 | 0.98 (0.95-1.00) | 1.029% | 0.104 |  | 1 | 0.99 (0.97-1.01) | 1.082% | 0.297 |
| Pt1e-04 | 32 | 1.01 (0.98-1.04) | 1.025% | 0.419 |  | 86 | 1.01 (0.99-1.03) | 1.081% | 0.488 |
| Pt0.001 | 257 | 1.03 (1.00-1.05) | 1.032% | 0.053 |  | 621 | 1.04 (1.02-1.06) | 1.095% | 2.67×10^-4^ |
| Pt0.05 | 6579 | 1.07 (1.04-1.10) | 1.073% | 1.35×10^-6^ |  | 16960 | 1.06 (1.04-1.08) | 1.116% | 6.40×10^-9^ |
| Pt0.1 | 11040 | 1.07 (1.04-1.10) | 1.075% | 7.27×10^-7^ |  | 28876 | 1.05 (1.03-1.07) | 1.110% | 1.49×10^-7^ |
| Pt0.2 | 18035 | 1.08 (1.05-1.11) | 1.088% | 3.08×10^-8^ |  | 47277 | 1.05 (1.03-1.07) | 1.107% | 4.25×10^-7^ |
| Pt0.3 | 23450 | 1.08 (1.05-1.11) | 1.094% | 7.49×10^-9^ |  | 61605 | 1.05 (1.03-1.07) | 1.110% | 1.21×10^-7^ |
| Pt0.4 | 27865 | 1.08 (1.05-1.11) | 1.095% | 5.72×10^-9^ |  | 72743 | 1.05 (1.03-1.07) | 1.111% | 6.25×10^-8^ |
| Pt0.5 | 31488 | 1.09 (1.06-1.12) | 1.104% | 6.46×10^-10^ |  | 82028 | 1.05 (1.03-1.07) | 1.110% | 1.36×10^-7^ |

OR were adjusting for birth year, sex, genotyping array, and ancestry principal components. Pt was p value cutoff thresholds. N_SNP was the number of SNP in the predictive model. R^2^ was Nagelkerke’s squared (R square). PRS = polygenic risk score. GWAS = Genome wide association study. PGC = Psychiatric Genomics Consortium.

**Table S6 Associations between polygenic risk scores (PRS, calculated using Clumping + Thresholding approach) for psychotic disorder and the risk of psychotic disorder at different p value thresholds**

| Pt | **GWAS summary statistics from UK Biobank base data** | | | |  | **GWAS summary statistics from PGC** | | | |
| --- | --- | --- | --- | --- | --- | --- | --- | --- | --- |
|  | N_SNP | OR (95% CI) | R^2^ | p value |  | N_SNP | OR (95% CI) | R^2^ | p value |
| Pt5e-08 | - | - | - | - |  | 114 | 1.12 (1.04-1.21) | 0.805% | 2.22×10^-3^ |
| Pt1e-06 | 1 | 0.99 (0.89-1.09) | 1.077% | 0.777 |  | 205 | 1.17 (1.08-1.26) | 0.874% | 4.84×10^-5^ |
| Pt1e-04 | 28 | 0.99 (0.90-1.10) | 1.076% | 0.906 |  | 1051 | 1.33 (1.23-1.43) | 1.241% | 9.06×10^-14^ |
| Pt0.001 | 225 | 1.06 (0.96-1.17) | 1.101% | 0.24 |  | 3149 | 1.40 (1.30-1.51) | 1.475% | 1.19×10^-19^ |
| Pt0.05 | 6250 | 1.07 (0.97-1.18) | 1.109% | 0.178 |  | 31177 | 1.63 (1.52-1.75) | 2.359% | 6.61×10^-42^ |
| Pt0.1 | 10723 | 1.11 (1.00-1.22) | 1.149% | 0.044 |  | 48527 | 1.61 (1.50-1.73) | 2.283% | 3.92×10^-40^ |
| Pt0.2 | 17781 | 1.09 (0.99-1.20) | 1.128% | 0.088 |  | 75841 | 1.62 (1.51-1.74) | 2.312% | 6.40×10^-41^ |
| Pt0.3 | 23327 | 1.10 (1.00-1.21) | 1.139% | 0.061 |  | 97814 | 1.64 (1.52-1.76) | 2.376% | 1.62×10^-42^ |
| Pt0.4 | 27751 | 1.08 (0.98-1.19) | 1.117% | 0.130 |  | 116375 | 1.64 (1.52-1.76) | 2.372% | 1.92×10^-42^ |
| Pt0.5 | 31465 | 1.08 (0.98-1.19) | 1.120% | 0.118 |  | 132043 | 1.63 (1.52-1.75) | 2.332% | 1.80×10^-41^ |

OR were adjusting for birth year, sex, genotyping array, and ancestry principal components. Pt was p value thresholds. N_SNP was the number of SNP in the predictive model. R^2^ was Nagelkerke’s squared (R square). PRS = polygenic risk score. GWAS = Genome wide association study. PGC = Psychiatric Genomics Consortium. – No results in this fields.

**Table S7 Associations between polygenic risk scores (PRS, calculated using Clumping + Thresholding approach) for stress-related disorder and the risk of stress-related disorder at different p value thresholds**

| Pt | **GWAS summary statistics from UK Biobank base data** | | | |  | **GWAS summary statistics from** **iPSYCH** | | | |
| --- | --- | --- | --- | --- | --- | --- | --- | --- | --- |
|  | N_SNP | OR (95% CI) | R^2^ | p value |  | N_SNP | OR (95% CI) | R^2^ | p value |
| Pt5e-08 | 0 | 0.89 (0.75-1.06) |  | 0.183 |  | 1 | 1.00 (0.92-1.09) | 1.689% | 0.967 |
| Pt1e-06 | 0 | 1.01 (0.90-1.14) | 2.634% | 0.837 |  | 3 | 0.96 (0.89-1.05) | 1.698% | 0.395 |
| Pt1e-04 | 33 | 1.01 (0.90-1.13) | 2.578% | 0.896 |  | 156 | 0.98 (0.90-1.06) | 1.692% | 0.607 |
| Pt0.001 | 218 | 0.92 (0.81-1.03) | 2.577% | 0.149 |  | 985 | 1.03 (0.95-1.12) | 1.696% | 0.447 |
| Pt0.05 | 6146 | 0.95 (0.84-1.07) | 2.629% | 0.378 |  | 22091 | 1.18 (1.09-1.28) | 1.876% | 8.37×10^-5^ |
| Pt0.1 | 10679 | 0.92 (0.82-1.04) | 2.596% | 0.182 |  | 37495 | 1.16 (1.07-1.26) | 1.842% | 3.78×10^-4^ |
| Pt0.2 | 17708 | 0.93 (0.82-1.04) | 2.621% | 0.199 |  | 62370 | 1.16 (1.07-1.26) | 1.839% | 4.28×10^-4^ |
| Pt0.3 | 23240 | 0.93 (0.83-1.05) | 2.618% | 0.236 |  | 82929 | 1.16 (1.07-1.26) | 1.845% | 3.24×10^-4^ |
| Pt0.4 | 27746 | 0.92 (0.82-1.04) | 2.612% | 0.170 |  | 100797 | 1.16 (1.07-1.26) | 1.848% | 2.84×10^-4^ |
| Pt0.5 | 31405 | 0.92 (0.82-1.04) | 2.624% | 0.175 |  | 115918 | 1.15 (1.06-1.25) | 1.831% | 6.10×10^-4^ |

OR were adjusting for birth year, sex, genotyping array, and ancestry principal components. Pt was p value thresholds. N_SNP was the number of SNP in the predictive model. R^2^ was Nagelkerke’s squared (R square). PRS = polygenic risk score. GWAS = Genome wide association study. – No results in this fields.

**Table S8 The association between polygenic risk scores (PRSs, calculated using Clumping + Thresholding approach) for psychiatric disorders and COVID-19**

| **Psychiatric disorders** | **Any COVID-19** | |  | **Severe COVID-19** | |
| --- | --- | --- | --- | --- | --- |
|  | **No. (%) of Any COVID-19** | **Odds Ratio** |  | **No. (%) of Severe COVID-19** | **Odds Ratio** |
|  |  | **(95% confidence interval)^a^** |  |  | **(95% confidence interval)^a^** |
| ***GWAS summary statistics from UK Biobank base data*** | | |  |  |  |
| **Substance misuse** | 10,868/143,562 (7.57%) | 1.03 (1.01-1.05) |  | 1,582/143,562 (1.10%) | 1.08 (1.03-1.13) |
| **Depression** |  | 1.03 (1.01-1.05) |  |  | 1.04 (0.99-1.09) |
| **Anxiety** |  | 1.03 (1.01-1.05) |  |  | 1.03 (0.98-1.09) |
| **Psychotic disorder** |  | 1.02 (1.00-1.04) |  |  | 1.00 (0.96-1.06) |
| **Stress related disorder** |  | 0.99 (0.97-1.01) |  |  | 0.98 (0.94-1.03) |
| ***GWAS summary statistics from publicly available GWAS summary statistics*** | | | | |  |
| **Substance misuse** | 21,736/287,123(7.57%) | 1.02 (1.01-1.04) |  | 3,146/287,123 (1.10%) | 1.03 (0.99-1.06) |
| **Depression** |  | 1.02 (1.00-1.03) |  |  | 1.03 (0.99-1.07) |
| **Anxiety** |  | 1.02 (1.00-1.03) |  |  | 1.05 (1.01-1.09) |
| **Psychotic disorder** |  | 1.01 (1.00-1.03) |  |  | 1.00 (0.97-1.04) |
| **Stress-related disorder** |  | 1.02 (1.01-1.04) |  |  | 1.08 (1.04-1.12) |

a Odd Ratio and 95% CIs (per standard deviation increase in the corresponding PRS) were estimated by logistic regression models, adjusting for birth year, sex, genotyping array, and ancestry principal components

**Table S9 The association between polygenic risk scores (PRSs, calculated using Clumping + Thresholding approach) for psychiatric disorders under different p value thresholds and COVID-19**

| Pt | **Substance misuse** | | | **Depression** | | | **Anxiety** | | | **Psychotic disorder** | | | **Stress-related disorder** | | |
| --- | --- | --- | --- | --- | --- | --- | --- | --- | --- | --- | --- | --- | --- | --- | --- |
|  |  | Any COVID-19 | Severe COVID-19 |  | Any COVID-19 | Severe COVID-19 |  | Any COVID-19 | Severe COVID-19 |  | Any COVID-19 | Severe COVID-19 |  | Any COVID-19 | Severe COVID-19 |
|  | R^2^ | OR (95% CI) | OR (95% CI) ^a^ | R^2^ | OR (95% CI) ^a^ | OR (95% CI) ^a^ | R^2^ | OR (95% CI) ^a^ | OR (95% CI) ^a^ | R^2^ | OR (95% CI) ^a^ | OR (95% CI) ^a^ | R^2^ | OR (95% CI) ^a^ | OR (95% CI) ^a^ |
| ***GWAS summary statistics from UK Biobank base data*** | | | | | | | | | | | | |  |  |  |
| Pt1e-06 | 1.698% | 1.00(0.98-1.02) | 1.00(0.95-1.05) | 1.699% | 1.01(0.99-1.03) | 1.00(0.95-1.05) | 1.698% | 1.00(0.98-1.02) | 1.00(0.95-1.05) | 1.700% | 0.99(0.97-1.01) | 1.02(0.97-1.07) | - | - | - |
| Pt1e-04 | 1.699% | 1.01(0.99-1.03) | 1.00(0.95-1.05) | 1.698% | 1.00(0.98-1.02) | 1.00(0.95-1.05) | 1.700% | 1.01(0.99-1.03) | 0.99(0.94-1.04) | 1.698% | 0.99(0.97-1.01) | 0.96(0.91-1.00) | 1.698% | 1.00(0.98-1.02) | 1.01(0.96-1.06) |
| Pt0.001 | 1.701% | 1.02(1.00-1.04) | 1.00(0.95-1.05) | 1.705% | 1.02(1.00-1.04) | 1.03(0.98-1.08) | 1.699% | 1.01(0.99-1.03) | 1.01(0.96-1.06) | 1.703% | 1.02(1.00-1.04) | 1.00(0.96-1.06) | 1.698% | 1.00(0.98-1.02) | 1.01(0.97-1.07) |
| Pt0.05 | 1.704% | 1.02(1.00-1.04) | 1.06(1.00-1.11) | 1.712% | 1.03(1.01-1.05) | 1.04(0.99-1.09) | 1.702% | 1.02(1.00-1.04) | 1.03(0.98-1.08) | 1.699% | 1.01(0.99-1.03) | 0.99(0.94-1.04) | 1.698% | 1.01(0.99-1.03) | 0.97(0.93-1.02) |
| Pt0.1 | 1.708% | 1.03(1.01-1.05) | 1.07(1.01-1.12) | 1.708% | 1.03(1.01-1.05) | 1.05(1.00-1.11) | 1.704% | 1.02(1.00-1.04) | 1.04(0.99-1.09) | 1.700% | 1.01(0.99-1.03) | 0.99(0.94-1.04) | 1.698% | 1.00(0.98-1.02) | 0.99(0.94-1.04) |
| Pt0.2 | 1.708% | 1.03(1.01-1.05) | 1.07(1.02-1.12) | 1.707% | 1.03(1.01-1.05) | 1.04(0.99-1.09) | 1.705% | 1.02(1.00-1.04) | 1.03(0.98-1.09) | 1.698% | 1.00(0.98-1.02) | 0.98(0.94-1.03) | 1.698% | 1.00(0.98-1.01) | 0.98(0.94-1.04) |
| Pt0.3 | 1.709% | 1.03(1.01-1.05) | 1.08(1.03-1.14) | 1.707% | 1.03(1.01-1.05) | 1.03(0.98-1.08) | 1.704% | 1.02(1.00-1.04) | 1.03(0.98-1.08) | 1.698% | 1.01(0.99-1.03) | 0.99(0.94-1.04) | 1.699% | 0.99(0.97-1.01) | 0.98(0.94-1.03) |
| Pt0.4 | 1.708% | 1.03(1.01-1.05) | 1.08(1.02-1.13) | 1.709% | 1.03(1.01-1.05) | 1.04(0.99-1.09) | 1.706% | 1.03(1.01-1.05) | 1.03(0.98-1.09) | 1.698% | 1.01(0.99-1.03) | 0.98(0.93-1.03) | 1.698% | 0.99(0.97-1.01) | 0.98(0.94-1.03) |
| Pt0.5 | 1.711% | 1.03(1.01-1.05) | 1.08(1.03-1.13) | 1.708% | 1.03(1.01-1.05) | 1.05(0.99-1.10) | 1.706% | 1.03(1.01-1.05) | 1.03(0.98-1.08) | 1.698% | 1.00(0.98-1.02) | 0.98(0.93-1.02) | 1.698% | 0.99(0.97-1.01) | 0.98(0.93-1.03) |
| ***GWAS summary statistics from publicly available GWAS*** | | | | | | |  |  |  |  |  |  |  |  |  |
| Pt5e-08 | - | - | - | 1.786% | 0.99(0.98-1.01) | 1.01(0.97-1.04) | 1.785% | 1.00(0.99-1.02) | 0.98(0.94-1.01) | 1.785% | 1.00(0.98-1.01) | 0.97(0.94-1.01) | 1.790% | 1.02(1.01-1.03) | 1.06(1.02-1.10) |
| Pt1e-06 | 1.785% | 1.00(0.99-1.02) | 1.03(1.00-1.07) | 1.785% | 1.00(0.98-1.01) | 1.01(0.98-1.05) | 1.785% | 1.00(0.99-1.02) | 0.98(0.94-1.01) | 1.785% | 1.00(0.98-1.01) | 0.97(0.94-1.01) | 1.788% | 1.01(1.00-1.03) | 1.02(0.99-1.06) |
| Pt1e-04 | 1.786% | 1.01(0.99-1.02) | 1.01(0.97-1.04) | 1.785% | 1.00(0.99-1.01) | 1.01(0.97-1.05) | 1.786% | 1.01(1.00-1.02) | 1.02(0.99-1.06) | 1.785% | 1.00(0.98-1.01) | 0.98(0.95-1.02) | 1.785% | 1.00(0.99-1.02) | 1.04(1.00-1.08) |
| Pt0.001 | 1.786% | 1.01(1.00-1.02) | 1.01(0.97-1.04) | 1.787% | 1.01(1.00-1.03) | 1.02(0.98-1.06) | 1.785% | 1.00(0.99-1.01) | 1.04(1.01-1.08) | 1.785% | 1.00(0.99-1.01) | 1.01(0.97-1.04) | 1.787% | 1.01(1.00-1.03) | 1.05(1.02-1.09) |
| Pt0.05 | 1.792% | 1.02(1.01-1.04) | 1.03(0.99-1.06) | 1.786% | 1.01(1.00-1.02) | 1.03(1.00-1.07) | 1.788% | 1.01(1.00-1.03) | 1.06(1.02-1.10) | 1.787% | 1.01(1.00-1.03) | 1.01(0.97-1.04) | 1.787% | 1.01(1.00-1.03) | 1.07(1.03-1.11) |
| Pt0.1 | 1.790% | 1.02(1.00-1.03) | 1.04(1.01-1.08) | 1.787% | 1.01(1.00-1.03) | 1.03(1.00-1.07) | 1.789% | 1.02(1.00-1.03) | 1.05(1.01-1.09) | 1.787% | 1.01(1.00-1.03) | 1.01(0.97-1.04) | 1.787% | 1.01(1.00-1.03) | 1.07(1.04-1.11) |
| Pt0.2 | 1.789% | 1.02(1.00-1.03) | 1.02(0.99-1.06) | 1.789% | 1.02(1.00-1.03) | 1.03(1.00-1.07) | 1.788% | 1.02(1.00-1.03) | 1.06(1.02-1.10) | 1.788% | 1.01(1.00-1.03) | 1.00(0.97-1.04) | 1.790% | 1.02(1.01-1.03) | 1.08(1.04-1.12) |
| Pt0.3 | 1.789% | 1.02(1.00-1.03) | 1.02(0.99-1.06) | 1.789% | 1.02(1.00-1.03) | 1.03(1.00-1.07) | 1.789% | 1.02(1.00-1.03) | 1.06(1.02-1.10) | 1.788% | 1.02(1.00-1.03) | 1.01(0.97-1.04) | 1.790% | 1.02(1.01-1.03) | 1.08(1.04-1.12) |
| Pt0.4 | 1.788% | 1.02(1.00-1.03) | 1.02(0.98-1.06) | 1.789% | 1.02(1.00-1.03) | 1.03(1.00-1.07) | 1.788% | 1.01(1.00-1.03) | 1.06(1.02-1.10) | 1.788% | 1.02(1.00-1.03) | 1.00(0.97-1.04) | 1.792% | 1.02(1.01-1.04) | 1.08(1.04-1.12) |
| Pt0.5 | 1.788% | 1.02(1.00-1.03) | 1.02(0.99-1.06) | 1.790% | 1.02(1.00-1.03) | 1.03(0.99-1.07) | 1.788% | 1.02(1.00-1.03) | 1.06(1.02-1.10) | 1.788% | 1.02(1.00-1.03) | 1.00(0.97-1.04) | 1.793% | 1.02(1.01-1.04) | 1.08(1.04-1.12) |

a Odd Ratio and 95% CIs (per standard deviation increase in the corresponding PRS) were estimated by logistic regression models, adjusting for birth year, sex, genotyping array, and ancestry principal components. Pt: p value threshold

Included participants

346,502

97,059,328

Restricted to the autosomal biallelic SNPs and imputation accuracy score > 0·1

SNPs with call rate > 98%, a minor allele frequency > 0·01, and Hardy–Weinberg equilibrium (p < 10^−6^)

Participants with genotyping rate > 98%

Participants with heterozygosity within ±3 SD from mean, without related individuals (i.e., kinship coefficients < 0.044)

**Participants and SNPs passed GWAS QC steps**

287,123

**287,123**

4,813,217

86,178,275

**4,813,217**

**SNPs**

**Participants**

346,502

**Figure S1 Flowchart of the entire GWAS quality control process**


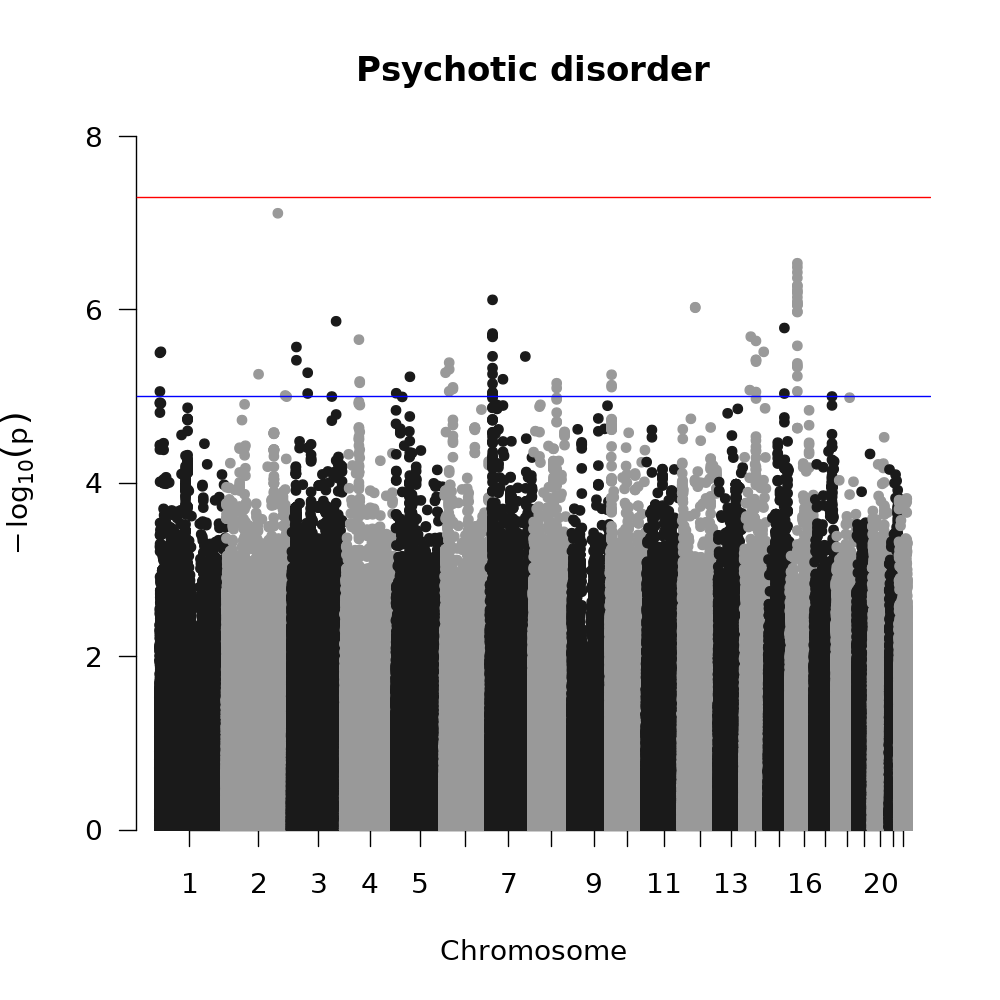


**A Substance misuse**

**B Depression**

**C Anxiety**

**D Psychotic disorder**

**E Stress-related disorder**


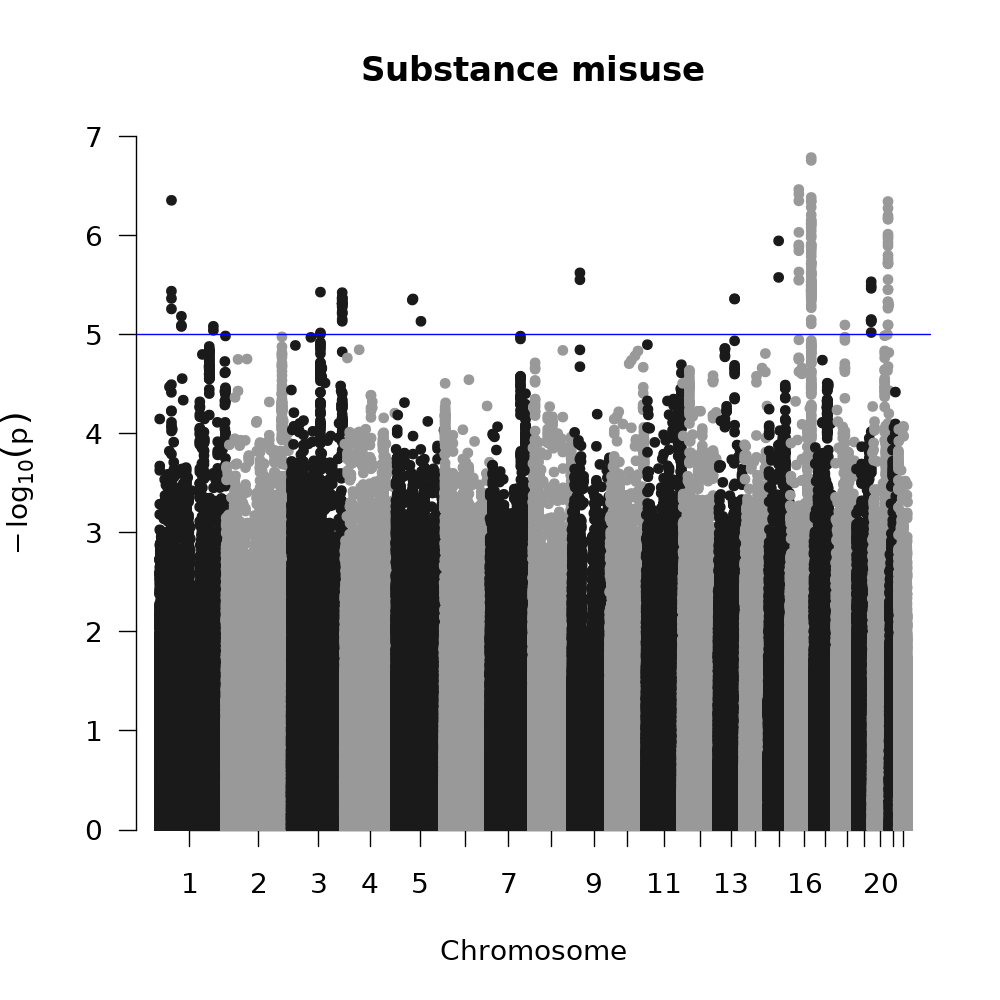

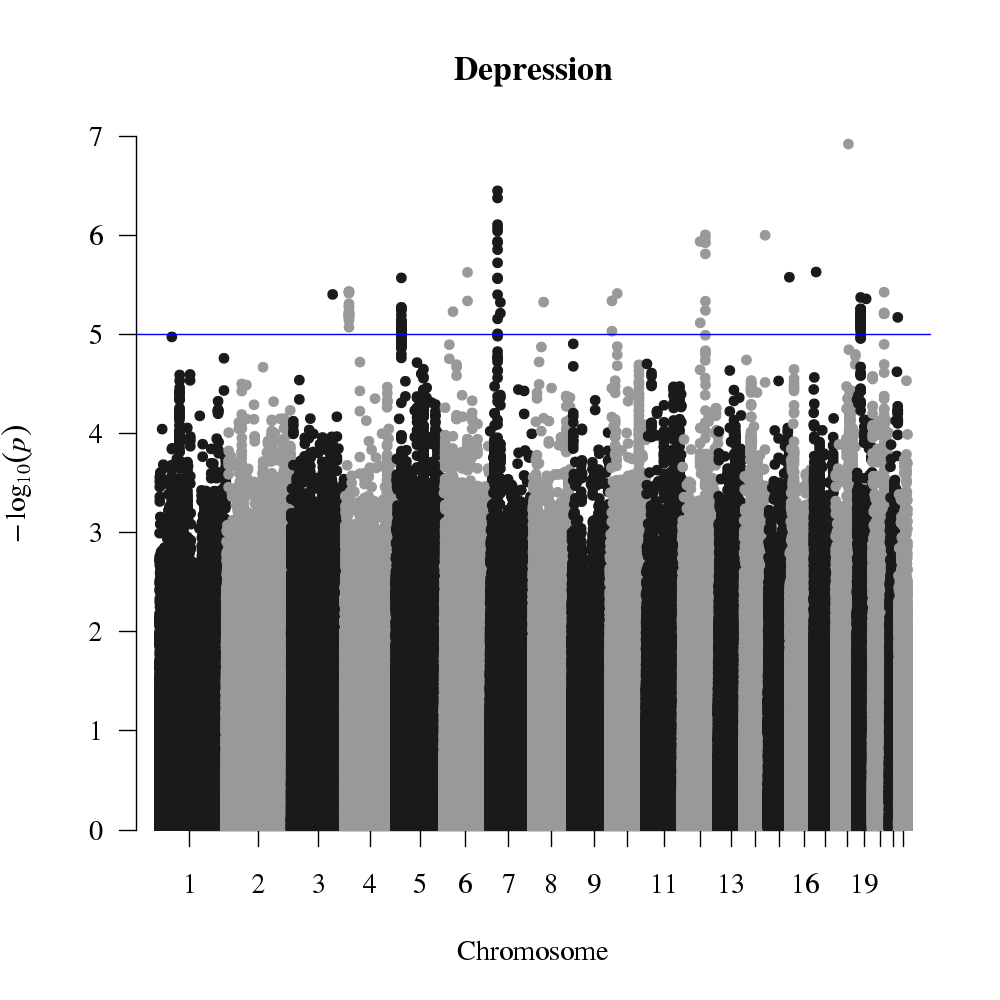

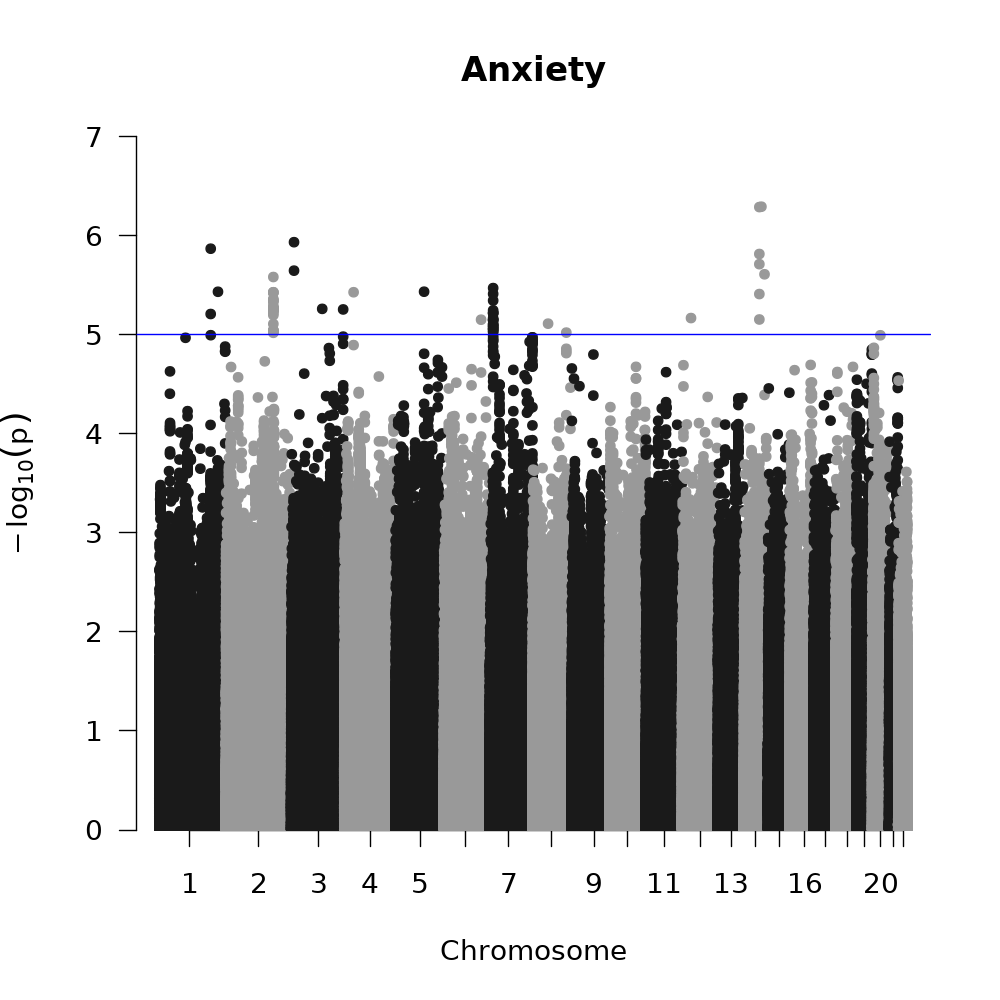

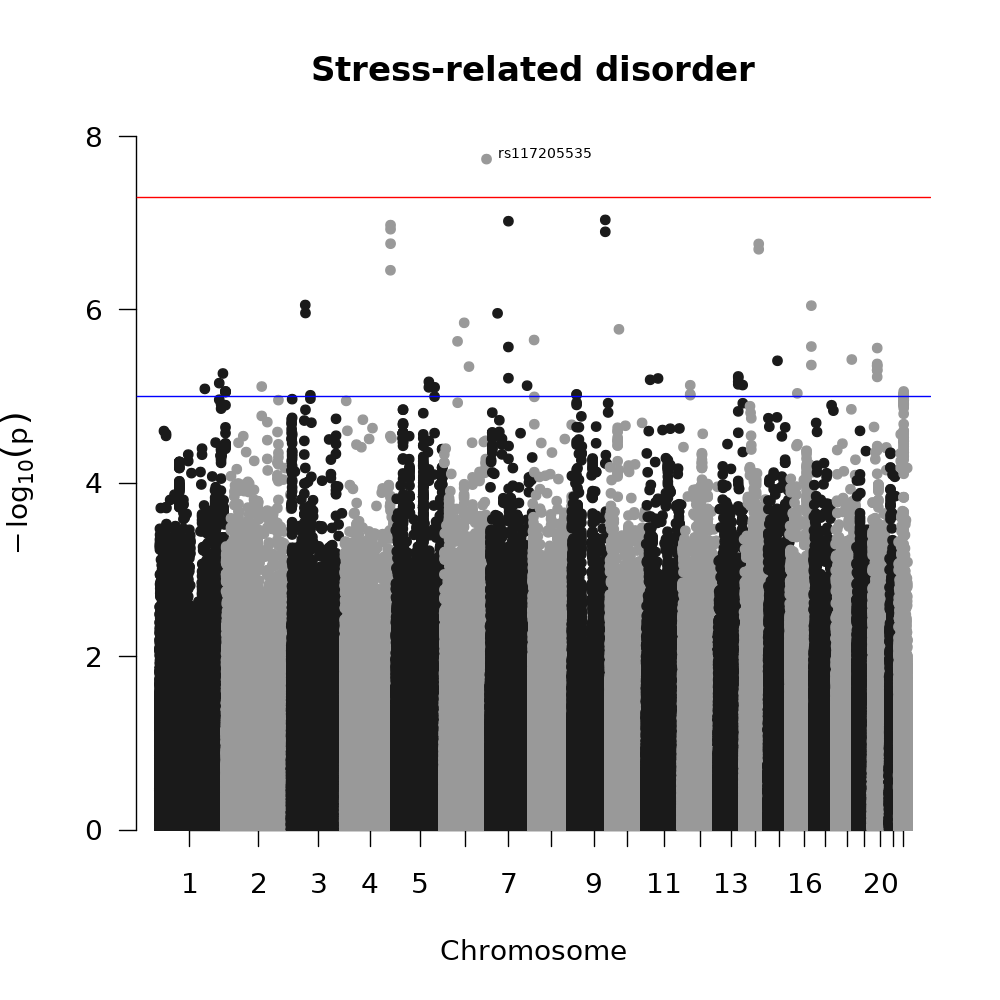


**Figure S2 Manhattan of the SNP-Based GWAS of psychiatric disorders, using UK Biobank base data (**SNP=single-nucleotide polymorphism)

**Figure S3 The association between categorized polygenic risk scores (PRSs, calculated using Clumping + Thresholding approach) for psychiatric disorders and COVID-19 risk ^a^**

^a^ Odd Ratio and 95% CIs were estimated by logistic regression models, adjusting for birth year, sex, genotyping array, and ancestry principal component.
